# Expression of the CGRP Family of Neuropeptides and their Receptors in the Trigeminal Ganglion

**DOI:** 10.1007/s12031-020-01493-z

**Published:** 2020-02-22

**Authors:** Lars Edvinsson, Anne-Sofie Grell, Karin Warfvinge

**Affiliations:** 1 Department of Internal Medicine, Lund University Hospital, Lund, Sweden; 2 Department of Clinical Experimental Research, Glostrup Research Institute, Rigshospitalet Glostrup Hospital, Copanhagen, Denmark

**Keywords:** CGRP peptides family, Ligands, Receptors, Immunohistochemistry, qPCR, mRNA analysis

## Abstract

The calcitonin gene-related peptide (CGRP) family of neuropeptides, consists of CGRP, adrenomedullin, amylin, and calcitonin. The receptors consist of either calcitonin receptor-like receptor (CLR) or calcitonin receptor (CTR) which for function needs an accessory protein, receptor activity-modifying proteins (RAMPs). CGRP has a pivotal role in primary headaches but the role of the other members of the CGRP family of peptides in headaches is not known. Here, we describe the expression of these molecules in the trigeminal ganglion (TG) to understand more on their possible role(s). Single or double immunohistochemistry were applied on frozen sections of rat TG using primary antibodies against CGRP, procalcitonin, calcitonin, adrenomedullin, amylin, RAMP1/2/3, CLR, and CTR. In addition, mRNA expression was measured by quantitative qPCR on TGs. CGRP and calcitonin showed rich expression in the cytoplasm of small to medium-sized neurons, and co-localized sometimes. Procalcitonin was observed in the glial cells. Immunoreactive fibers storing both CGRP and calcitonin were also observed. Adrenomedullin immunoreactivity was found in the satellite glial cells and in fibers, probably the myelinating Schwann cells. Amylin was found in the cytoplasm in many TG neurons. Levels of mRNA expression for adrenomedullin, amylin, CLR, RAMP1, RAMP2, RAMP3, and CTR were measured using qPCR. The experiments verified the expression of mRNA in the TG with the exception of CTR, which was above the limit of detection indicating little or no mRNA expression. In addition to the well-known CGRP receptor (CLR/RAMP1) and the receptor for calcitonin—CTR, we propose that other receptors exist in the rat TG: adrenomedullin receptor AM_2_ (CLR/RAMP3) in mainly the satellite glial cells, amylin receptors AMY_1_ (CTR/RAMP1) in mainly neurons, and AMY_3_ (CTR/RAMP3) in the satellite glial cells. It is important to compare peptides and receptors side-by-side in studies to help address questions of actions resulting from cross-reactivity between receptors. Several of the diverse biological actions of the CGRP family of peptides are clinically relevant. Our findings demonstrate the specific ligand and receptor sites in the rat trigeminal ganglion, highlighting recognition mechanisms to facilitate drug development.

## Introduction

The trigeminal ganglion (TG) is primarily a sensory ganglion of the trigeminal nerve (the V^th^ cranial nerve) that occupies Meckel’s cave at the base of the brain and is surrounded by the dura mater. The neurons within the TG are firmly enveloped by the satellite glial cells (SGCs), demonstrating the close interaction between the neurons and glial cells coupled by gap junctions (Hanani 2005). TG is linked peripherally to the ophthalmic, maxillary and part of the mandibular nerves, and centrally to the trigeminal nucleus caudalis (TNC) and dorsal root C_1_-C_3_ (Edvinsson 2011).

Calcitonin gene-related peptide (CGRP) is richly located in numerous sites throughout the central and peripheral nervous systems (Russell et al. 2014; Warfvinge and Edvinsson 2019a). A sizeable population of CGRP neurons within the TG signifies a major role for CGRP in trigeminal transmission. About half of all neurons in the TG express CGRP which is visualized by immunohistochemical staining with CGRP antibodies and by in situ hybridization to localize mRNA for CGRP (Eftekhari et al. 2010; Miller et al. 2016). CGRP-positive neurons are predominantly of unmyelinated small-medium diameter which is indicative of cell bodies of C-type sensory pain fibers (Eftekhari et al. 2013). The human CALCA gene codes for both calcitonin and αCGRP via an alternative splicing mechanism in neural tissues (Amara et al. 1982). Both calcitonin and αCGRP are cleaved from larger pro-peptides known as procalcitonin (Pro-CT) and pro-CGRP (Buervenich et al. 2001). Research on primary headaches has revealed an important role of CGRP mainly related to the headache phase of a migraine attack, while the involvement and expression of other members of this family of peptides is less well known (Edvinsson et al. 2018).

The CGRP family of peptides shares structural homology and includes CGRP itself, calcitonin (CT), adrenomedullin (AM), and amylin (AMY). They have a widespread distribution throughout the body with particular abundance in the brain, the gastrointestinal system, and in various parts of the circulation (Russell et al. 2014). The members of the CGRP family might hypothetically be clinically relevant drug targets due to their roles in the regulation of several critical homeostatic processes (Hendrikse et al. 2018).

The peptides are ligands for closely related family B of G protein-coupled receptors (GPCRs), and they share structural homology (Hay et al. 2014). The peptides activate GPCRs which can heterodimerize with accessory proteins called receptor activity-modifying proteins (RAMPs). The RAMPs, a small family of three proteins, are single transmembrane-spanning proteins (TM) which may alter the pharmacology, functionality, and cell trafficking of these specific GPCRs (McLatchie et al. 1998). Currently, the most central is the seven transmembrane (TM7) complex, calcitonin receptor-like receptor (CLR) (McLatchie et al. 1998). CLR is a required element of receptors for CGRP and adrenomedullin. To form a functional receptor, CLR has to form a complex with RAMP1–3 to create these receptors (Table 1) (Hay et al. 2018). AMY receptors comprise a core family B of GPCR formed by the CT receptor (CTR) with RAMP1 and RAMP3 (Table 1). The CTR is also able to act as a receptor by itself. The other defined receptors are heteromeric and are composed of CTR in association with one of the RAMPs (Poyner et al. 2002). Due to the complexity of this peptide-receptor system (Table 1), their expression in the trigeminal system is not yet clear and their possible functional roles are yet to discover.

**Table 1:** Interaction of CLR and CTR with RAMP1, RAMP2, and RAMP3 and relative potency of CGRP, CT, AM, or AMY of the appropriate receptor

Ligands	Receptor components	Ligands relative potency to the receptor
CGRP	CLR + RAMP1	CGRP > AM
Adrenomedullin (AM)	CLR + RAMP2	AM > AM2
Adrenomedullin2 (AM2)	CLR + RAMP3	AM = AM2
Calcitonin (CT)	CTR	CT > AMY
Amylin1 (AMY)	CTR + RAMP1	CGRP = AMY
Amylin3 (AMY3)	CTR + RAMP3	AMY

The first study of CGRP distribution in the TG was performed in the cat in 1985 (Uddman et al. 1985). More recent work has in detail described the distribution of CGRP and its receptor components in the rat and human TG (Eftekhari et al. 2010) and in the rat retina (Blixt et al. 2017). From these studies, we conclude that almost all of the CGRP-negative neurons contain CGRP receptor components CLR and RAMP1. In addition, all RAMP1 immunoreactive cells co-expressed CLR (Blixt et al. 2017), and therefore, we proposed that RAMP1 expression primarily demonstrates the functional CGRP receptor.

The present study was designed to define the expression of the CGRP family of peptides and their receptor components in the TG in order to lay the foundation to a better understanding of their respective physiology, pathophysiology, and possible therapeutic potential (Hendrikse et al. 2018). It is particularly relevant to investigate the trigeminal system because of its central role in migraine pathophysiology. Here, we describe the expression and localization of the CGRP family of peptides and their receptors in the rat TG by using immunohistochemistry and quantitative PCR (qPCR).

## Material and Methods

### Animals

Immunohistochemistry followed the guidelines from the Regional Ethical Review Board in Lund, Sweden (M17–15). Quantitative PCR (qPCR) followed the guidelines from the European Community Council directive (2010/63/EU) for Protection of Vertebrate Animals Used for Experimental and other Scientific Purposes.

Animals were housed in the local animal facility in a temperature- (22–23 °C) and humidity-controlled environment with 12 h light and 12 h dark cycle and ad libitum access to standard chow and water.

### Immunohistochemistry

Male Sprague-Dawley rats (*n* = 10, 250–300 g) were sedated using dry ice (CO_2_). Shortly thereafter, the rats were decapitated, and the TGs were carefully dissected out. TGs were submerged in 4% formaldehyde in PBS buffer for 4 h. Subsequently, TGs were then washed in rising concentration of 10% and 25% of sucrose in Sorensen’s phosphate buffer (pH 7.2) to ensure cryoprotection. Finally, TGs were embedded in a gelatin medium (30% egg albumin, 3% gelatin) and stored at − 20 °C.

Ten micrometer cryosections were washed in PBS with 0.25% Triton (PBS-T) for 15 min. Next, primary antibodies were applied (Table 2). The sections were incubated in an incubation chambers at + 8 °C overnight and, during the following day, the glass slides were submerged in PBS-T 2 × 15 min. The remaining experiment was completed in a dark room, in order to preserve the fluorescence of the secondary antibodies. Appropriate secondary antibodies were diluted according to manufacturer’s instructions and incubated for 1 h at room temperature (Table 2). Next, the sections were washed in PBS-T for 2 × 15 min and mounted with Vectashield mounting medium containing 4′, 6-diamino-2-phenylindole (DAPI, Vector Laboratories, Burlingame CA, USA).

**Table 2:** Details of primary and secondary antibodies

Primary antibodies				
Antigen (species)	Dilution	Detects	Supplier	Reference
CGRP (guinea pig) PA1–36017	1:500	Synthetic human CGRP	Thermo Scientific, IL, USA	Eftekhari S, Salvatore CA, Gaspar RC, Roberts R, O’Malley S, Zeng Z, Edvinsson L. 2013 Dec;12(6):937–49.
Calcitonin (mouse), NBP1–30051	1:50	Calcitonin	Novus Biologicals, CO, USA	Boess F, Bertinetti-Lapatki C, Zoffmann S et al. J Mol Endocrinol 2013 Apr 12 [PMID: 23463748]
Adrenomedullin (mouse) sc-8462	1:100	Adrenomedullin of human origin	Santa Cruz Biotechnology, CA, USA	Kastin, A.J., Akerstrom, V., Hackler, L. and Pan, W. 2001. Horm. Metab. Res. 33: 19–2
Amylin (rabbit), PA5–32261	1:100	Amylin	Thermo Scientific, IL, USA	None available
Procalcitonin (rabbit), AbCam 53897	1:500	Synthetic human procalcitonin	Abcam, Cambridge Biomedical Campus, Cambridge, CB2 0AX, UK	Tajti, J., Kuris, A., Vecsei, L., Xu, C.B., and Edvinsson, L. (2011). Cephalalgia *31*, 95–105.
CLR (rabbit)	1:500	C-terminal of rat CLR	Merck & Co., Inc., USA	Eftekhari S, Salvatore CA, Calamari A, Kane SA, Tajti J, Edvinsson L.Neuroscience. 2010 Aug 25;169(2):683–96.
RAMP1–844 (goat)	1:100	C-terminal of human RAMP1	Merck & Co., Inc., USA	Eftekhari S, Salvatore CA, Calamari A, Kane SA, Tajti J, Edvinsson L.Neuroscience. 2010 Aug 25;169(2):683–96
RAMP2 (rabbit) GTX108524-S	1:100	RAMP2 protein	GeneTex, USA	Jan Blom, Thomas J. Giove, Winnie W. Pong, Todd A. Blute, William D. Eldred. Molecular Vision 2012; 18:1339–1353
RAMP3 (mouse) sc-365313	1:100	RAMP3 of mouse, rat and human	Santa Cruz Biotechnology, CA, USA	McLatchie LM^1^, Fraser NJ, Main MJ, Wise A, Brown J, Thompson N, Solari R, Lee MG, Foord SM. Nature. 1998 May 28;393(6683):333–9.
CTR (rabbit), CAU24308	1:100	Calcitonin receptor of rat	BioMatik, Delaware, USA	None available
Secondary antibodies				
	Dilution	Filter	Supplier	
Anti-guinea pig	1:100	Alexa 488	Thermo Scientific, IL, USA	
Anti-mouse	1:400	Cy3	Jackson Immunoresearch, PA, USA	
Anti-mouse	1:100	Alexa 488	Thermo Scientific, IL, USA	
Anti-rabbit	1:400	Cy3	Jackson Immunoresearch, PA, USA	
Anti-rabbit	1:100	Cy2	Jackson Immunoresearch, PA, USA	
Anti-goat	1:400	Cy3	Jackson Immunoresearch, PA, USA	
Anti-goat	1:100	C2	Jackson Immunoresearch, PA, USA	

Each procedure was repeated minimum three times to validate the results and to minimize any experimental errors.

To determine which combination of antibodies that resulted in the most clear and unambiguous outcome, a series of different peptides and receptor primary antibodies were matched with various secondary antibodies, and their respective combinations were investigated. The present procedure will only include the antibodies used to produce consistent and comparable results. Antibodies are summarized and distinguished in Table 1. The development and specificity of CLR and RAMP1 antibodies have been demonstrated in a study at our laboratory by Eftekhari et al. (2010)), where the specificity of the antibodies was confirmed in HEK293 cells stably expressing the CGRP receptor and by Western blot. Pre-absorption controls also have been performed with the CGRP, CLR, and RAMP1 antibodies (Eftekhari et al. 2010).

For double immunohistochemistry, the procedure was repeated two consecutive times. The first primary antibody was matched with its appropriate secondary antibody before the second round of primary antibodies was applied and finally mounted. Negative controls were performed for each set by omitting the primary antibody. Any resulting immunofluorescence would suggest unspecific binding of the secondary antibodies.

**Fig. 1:**
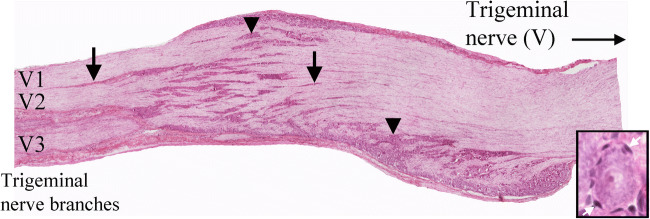
HTX staining or rat TG. TG is a sensory ganglion of the trigeminal nerve (the V^th^ cranial nerve), and that gives off the ophthalmic (V1), maxillary (V2), and part of the mandibular (V3) nerves. Sensory information arrives first at the brainstem and upper spinal cord, then further processed in thalamus and cortex where conscious perception of pain occurs. TG contains the cell bodies for these sensory neurons. Within TG, clusters (arrow heads) and rows of neurons (arrows) were found and, in addition, nerve fibers proximal to the cell bodies. Moreover, the neurons were firmly enveloped by the SGCs, constituting > 90% of the cells. Insert shows a neuron enveloped by SGCs (white arrows)

In addition, cryosections were hematoxylin-eosin stained (Htx) (Fig. 1) using the following protocol: Htx (4 min), tap water, Eosin (1 min), distilled water, alcohol (70%, 95%, 99.5%), xylene, and mounted with xylene-based Pertex (HistoLab, Gothenburg, Sweden).

The stainings were examined using a light/epifluorescence microscope (Nikon 80i, Tokyo, Japan) combined with a Nikon DS-2MV camera. Images were analyzed and examined in Adobe Photoshop CS3 (v10.0 Adobe Systems, Mountain View, CA), and images taken with different filters were superimposed in order to determine potential co-localization.

### Quantitative Polymerase Chain Reaction

Six male Sprague-Dawley rats (250–350 g) were sedated with CO_2_ and euthanized by guillotining. Both left and right TGs were dissected and used for one sample. After dissection, the TGs were cut into smaller pieces with a scalpel and immediately frozen on dry ice. TG was homogenized on dry ice 6 × 20 s in lysing matrix D tubes containing 1.4 mm ceramic spheres (MP Biomedicals, USA) and lysis buffer (ML buffer) from the NucleoSpin miRNA isolation kit (Macherey-Nagel, Germany) using a FastPrep-24™ 5G instrument (MP Biomedicals, USA). The NucleoSpin miRNA isolation kit was used to extract total RNA according to the manufacturers’ protocol. Total RNA concentration was measured by the NanoDrop 2000 UV-Vis spectrophotometer (ThermoFisher Scientific, USA). A ratio of sample absorbance at 260 nm and 280 nm in the range of 1.9 to 2.2 was acceptable.

Two thousand nanograms RNA was synthesized to cDNA using the RT^2^ First-Strand Kit (Qiagen, USA) according to the manufacturer’s protocol. qPCR was performed in a 10-μl reaction volume containing RNAase free water, 20× TaqMan gene expression assay (ThermoFisher Scientific, USA), 2× TaqMan universal PCR master mix (ThermoFisher Scientific, USA), and 2 μl cDNA using the QuantStudio 12 K Flex real-time PCR system (ThermoFisher Scientific, USA) with ROX as a passive reference. The thermal cycling condition included an initial denaturation step at + 50 °C for 2 min and + 95 °C for 10 min followed by 45 PCR cycles at + 95 °C for 15 s and + 60 °C for 1 min. An inter-plate control was used for all TaqMan gene expression assays to compare the thermal cycling between plates. A no-template was used as negative control for all TaqMan gene expression assays where RNAse free water was added instead of cDNA. All TaqMan gene expression assays were pipetted in triplicates for each sample (Table 3): adrenomedullin (*Adm*) (Rn00562327_m1), amylin (*IAPP*) (Rn00561411_m1), calcitonin receptor-like, CLR (*Calcrl*) (Rn00562334_m1), RAMP1 (*Ramp1*) (Rn01427056_m1), RAMP2 (*Ramp2*) (Rn00824652_g1), RAMP3 (*Ramp3*) (Rn00571815_m1), and calcitonin receptor, CTR (*Calcr*) (Rn00587525_m1). All the TaqMan gene expression assays are commercially available at ThermoFisher Scientific and not custom-made.

**Table 3:** Gene expression of adrenomedullin (*Adm*), Amylin (*IAPP*), calcitonin receptor-like (*Calcrl*), calcitonin receptor (*Calcr*), receptor activity-modifying protein 1 (*Ramp1*), receptor activity-modifying protein 2 (*Ramp2*), and receptor activity-modifying protein 3 (*Ramp3*) in the trigeminal ganglion (TG). *n* is the number of rats

	TG	
	Mean ± SEM	*n*
*Adm*	30.8 ± 0.15	6
*IAPP*	30.0 ± 0.10	6
*Calcrl*	28.9 ± 0.12	6
*Calcr*	36.6 ± 0.14	6
*Ramp1*	27.9 ± 0.15	6
*Ramp2*	25.2 ± 0.07	6
*Ramp3*	26.5 ± 0.14	6

### Calculations and Statistics for Quantitative Polymerase Chain Reaction

The threshold cycle (C_t_) is the intersection between the amplification curve and the threshold line, and it was determined using the QuantStudio 12 K Flex software (ThermoFisher Scientific, USA). Technical triplicates of C_t_ were averaged for each TaqMan gene expression assay. C_t_ values are plotted on the y-axis using GraphPad Prism 8. The y-axis is log and reversed. Data is presented as mean ± standard error of the mean (SEM), and *n* refers to the number of rats.

We decided to show C_t_ values instead of ΔC_t_ values (C_t,sample_ − C_t,average of references_), due to the extremely low C_t_ value of *Calcr* in the TG, and because we want to show whether the gene expression is present or not.

## Results

### Morphology of the Trigeminal Ganglion (Fig. 1)

TG is a sensory ganglion of the trigeminal nerve (V) and is linked to the ophthalmic (V1), maxillary (V2), and part of the mandibular (V3) nerves. Within TG, there are clusters and rows of neurons and, in addition, nerve fibers proximal to the cell bodies. Moreover, the neurons are firmly enveloped by the SGCs, constituting > 90% of the number of cells.

### Ligands Expression (Fig. 2)

CGRP is expressed in the cytoplasm, in a granular pattern, in many neurons, mainly in small- to medium-sized neurons in a pattern in agreement with previous publications (Eftekhari et al. 2010; Lennerz et al. 2008; Miller et al. 2016). The cellular CGRP is often expressed in or close to the membranes of the Golgi apparatus (Warfvinge, unpublished). In addition, CGRP immunoreactivity was detected in varicose fibers that are of the C-type of sensory unmyelinated nerves, and which often in the literature is referred to as “pearl-like.” The Aδ-fibers, surrounded by myelinating Schwann cells, did not contain CGRP.

**Fig. 2:**
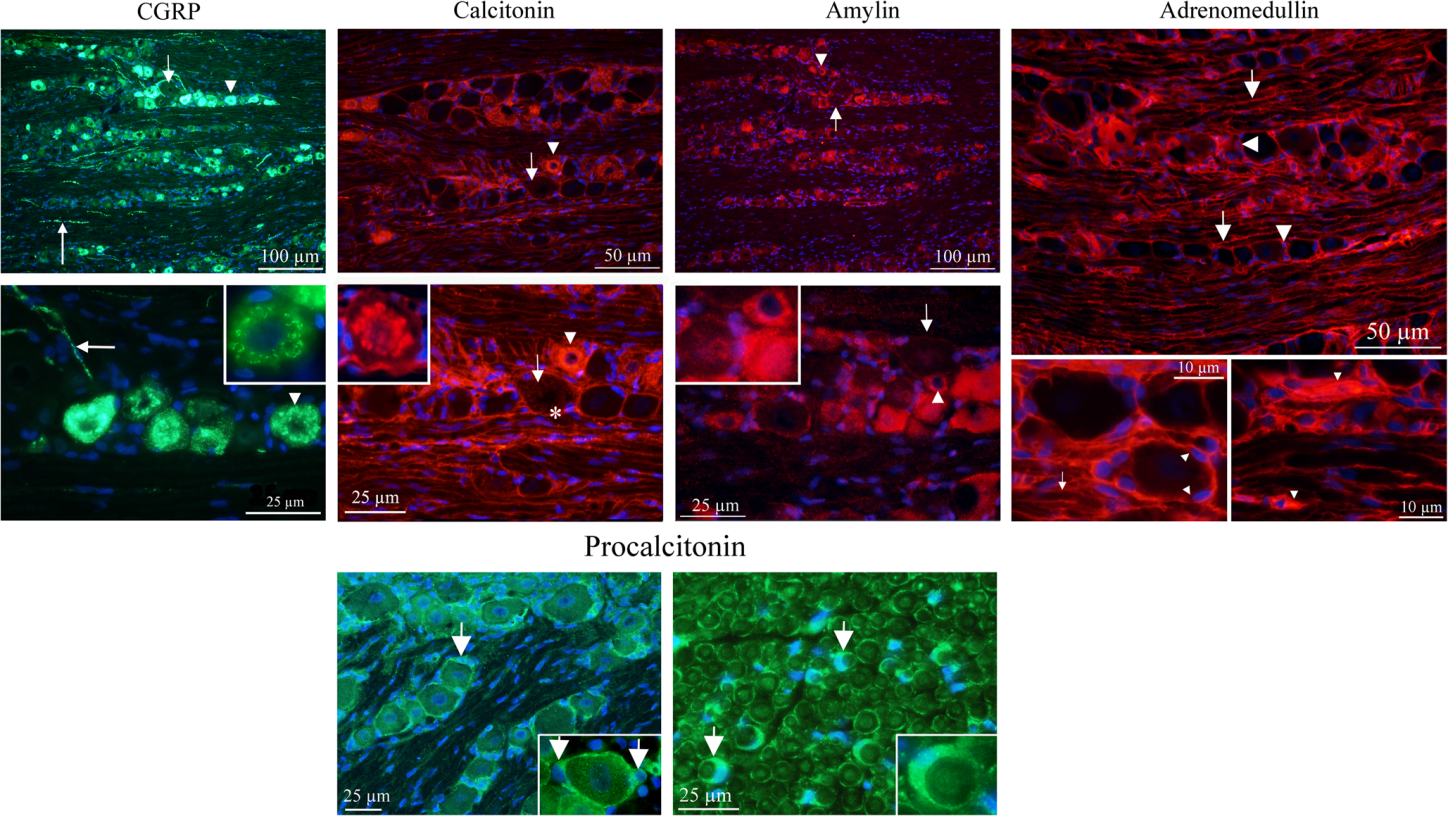
Ligand immunohistochemistry. **a** CGRP is expressed in a granular pattern in many neurons, mainly in small- to medium-sized neurons (arrow heads). The cellular CGRP is expressed in vesicles (insert). In addition, pearl-like CGRP immunoreactivity was detected in fibers that are of the C-type of sensory unmyelinated nerves (long arrows). The myelinated fibers do not contain CGRP. Short arrow points at a large negative neuron. CT immunoreactivity displayed a similar pattern as for CGRP; granular staining of small- to medium-sized neurons (arrow heads, insert) and pearl-like staining of fibers. Also, SGCs were CT immunoreactive (asterisks). Arrows point at a large negative neuron. AMY was exclusively expressed in the neurons, mainly small to medium sized (arrow heads). In some of the cells, the expression was granular, but in others, a general cytoplasmic immunoreactivity. Arrows point at a large negative neuron. Insert shows three different cells: one negative and two amylin immunoreactive. AM was expressed in the glial cells, both the SGCs (arrow heads) and cells enveloping the neuronal processes (arrows), probably myelinating cells. **b** Pro-CT was expressed exclusively in the glial cells. Panel to the left shows SGC staining (arrows) and panel to the right shows staining of the Schwann cells (arrows and insert) in a perpendicular cut of the trigeminal nerve

Neuronal soma CT expression displayed a similar pattern as that seen for CGRP (and was co-localized in some cells); granular staining of mainly small to medium-sized neurons and pearl-like staining of C-type fibers. The number of CT-positive cells and pearl-like-positive fibers were, however, less than those seen in CGRP immunohistochemical stained TGs. Yet, the main difference between CGRP and CT immunoreactivity was the CT cytoplasmic granular staining of SGCs, and of Aδ-fibers, which did not occur at CGRP immunohistochemistry.

Procalcitonin (Pro-CT) expression in TG has previously published (Tajti et al. 2011). We here confirm these results. Pro-CT immunoreactivity was observed exclusively in the glial cells, the SGCs, and the myelinating Schwann cells (Fig. 2b). Pro-CT immunoreactivity was found in the soma of the Schwann cells. The myelinated layers of the Schwann cells, surrounding the thick Aδ-fibers, did not express Pro-CT.

AMY was exclusively expressed in the cytoplasm of small- to medium-sized neurons. In some of the cells, the expression was granular, but in others, a general cytoplasmic immunoreactivity was observed.

AM was expressed in the thin cytoplasm of the glial cells, both the SGCs and cells enveloping the neuronal processes, probably Schwann cells. In addition, immunoreactivity was found in blood vessel walls, indicating vascular endothelial staining (not shown).

### Receptor Elements Expression (Fig. 3)

CLR and RAMP1 expression has earlier been examined in detail (Eftekhari et al. 2010; Lennerz et al. 2008; Miller et al. 2016). The present paper confirms these results. The receptor components were expressed in the cytoplasm of the neurons (mainly the larger ones) and the SGCs, and the thick fibers, typical for Aδ-fibers.

**Fig. 3:**
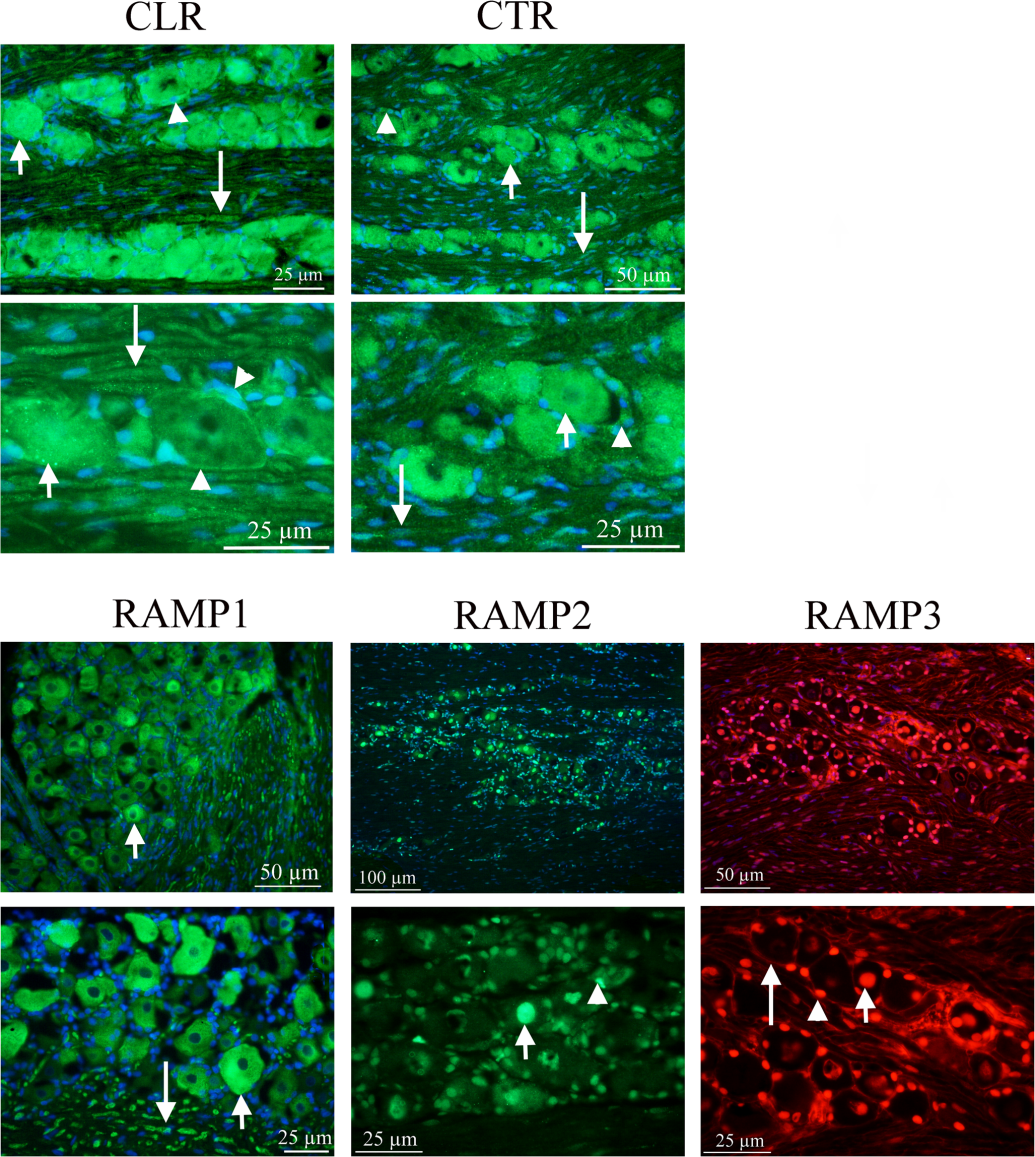
Receptor immunohistochemistry CLR and RAMP1 expression has earlier been examined in some detail (Eftekhari et al. 2010; Lennerz et al. 2008). The present paper confirm these results. The receptor components were expressed in neurons (mainly the larger ones, short arrows), in the SGCs (arrow heads) and the thick fibers, typical for Aδ-fibers (long arrows). RAMP2 and RAMP3 were expressed in the nuclei, both in those of neurons (arrows) and glia cell nuclei (arrow heads). In addition, RAMP3 was expressed in the glial cells (long arrow), however weaker than the nuclei staining. CTR was expressed in varying intensity in most neurons (short arrows) and SGCs (arrow heads). In addition, the nuclei of the glial cells surrounding the thick fibers showed immunoreactivity. The thick fibers also expressed CTR (long arrows), but weaker compared to CLR staining

RAMP2 and RAMP3 were expressed in the nuclei, both in those of neurons and of glia cells. In addition, RAMP3 was expressed in the cytoplasm of the glial cells (possibly both SGCs and Schwann cells enveloping the Aδ-fibers); however, the expression was considerably weaker than the nuclei staining.

CTR was expressed with varying intensity in the cytoplasm of the most neurons and SGCs. In addition, the nuclei of the glial cells (Schwann cells) surrounding the Aδ-fibers showed CTR immunoreactivity. In addition, the Aδ-fibers also expressed CTR, but in a weak manner.

### Ligand/Ligand Expression (Fig. 4)

To provide morphological clues for functionality of the CGRP family of peptides in the TG and cross-reactivity, i.e., selectivity of the antibodies used, we performed a series of double immunohistochemistry experiments. In these experiments, we suggest that areas with no co-localization represent areas with no cross-reactivity.

**Fig. 4:**
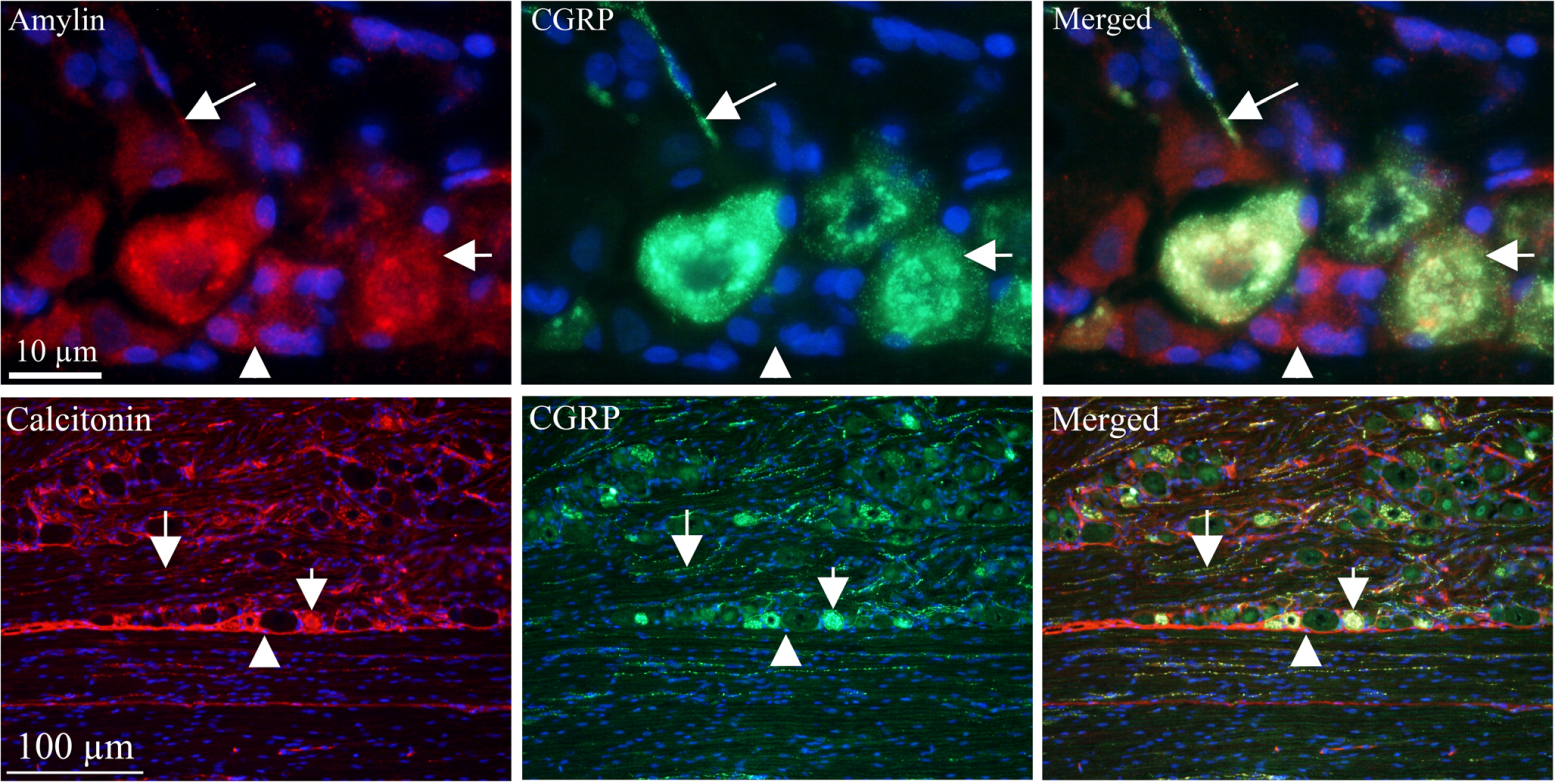
Ligand/ligand immunohistochemistry AMY and CGRP double staining showed co-expression in some of the small- to medium-sized neurons (short arrows) and thin C-fibers (long arrows); however, some were seen to only express CGRP or AMY (arrow heads). The number of CGRP-positive cells appeared to be more abundant than those storing AMY. Double staining with CT and CGRP antibodies showed co-expression in the small- to medium-sized neurons (short arrows) and in fibers (long arrows). In addition, CT was expressed in the SGCs (arrow heads)

Double staining with CGRP and CT antibodies showed co-expression in the cytoplasm of small-sized neurons. We accordingly suggest that this could indicate cross-reactivity between CGRP and CT antibodies. In addition, CT alone was expressed in the SGCs and thus CT expression in SGCs represent no cross-reactivity.

CGRP and AMY double staining showed co-expression in the cytoplasm of some of the small- to medium-sized neurons and in thin C-fibers; however, some were seen to only express CGRP and not AMY. The number of CGRP-positive cells appeared to be more abundant than those storing AMY.

AM was double stained with CGRP or AMY, respectively (not shown). No co-localization was found in neither with AM plus CGRP, nor with AM plus AMY double immunohistochemistry. AM and CT double staining was not performed since both antibodies were made in mouse.

### Receptor/Receptor Expression (Fig. 5)

RAMP1 and CLR are co-expressed mainly in the cytoplasm of larger neurons and in Aδ neuronal fibers (not shown).

**Fig. 5:**
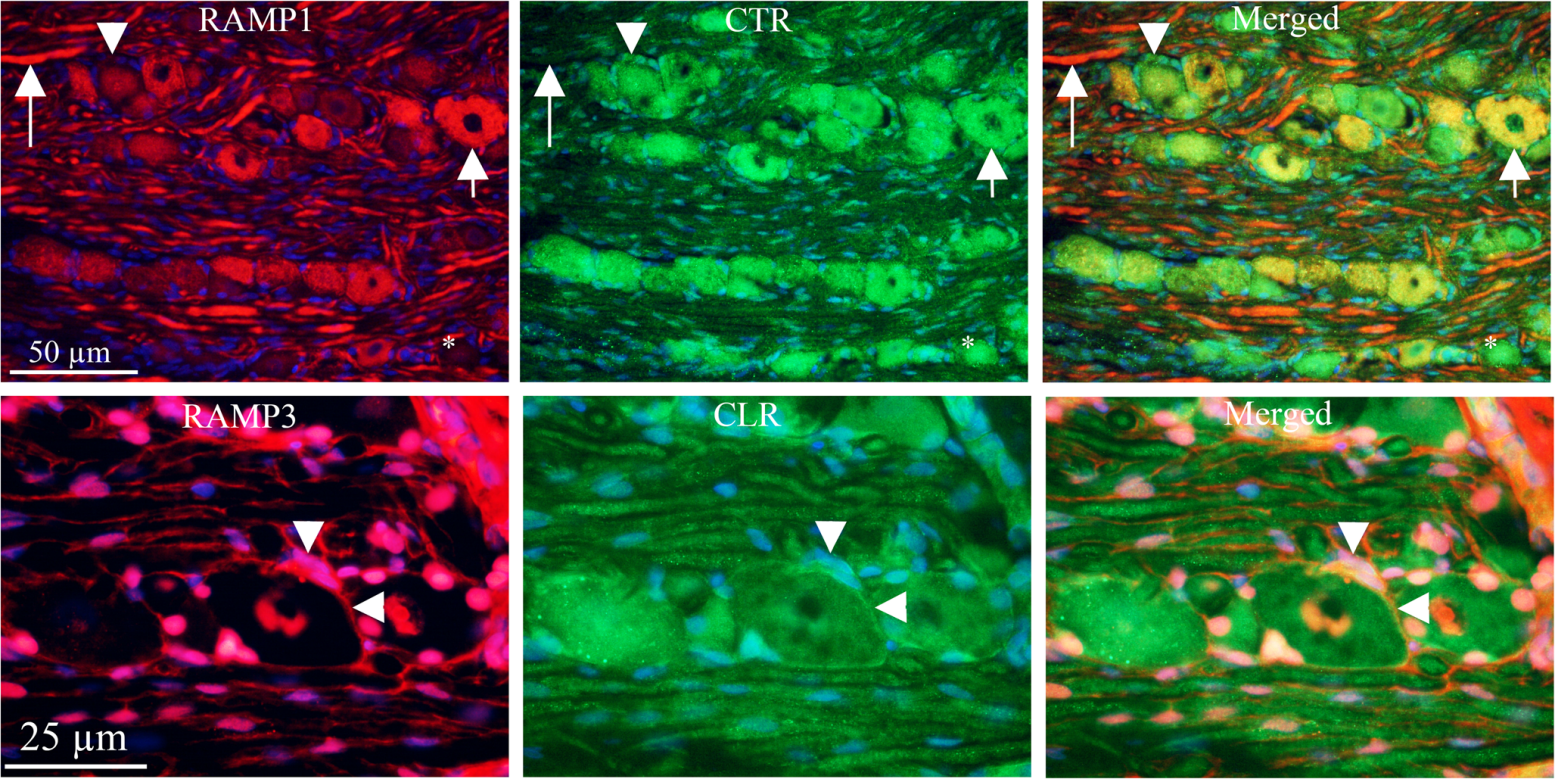
Receptor/receptor immunohistochemistry RAMP1 is expressed in neurons, in the SGCs and the thick fibers. CTR is expressed with varying intensity in most neurons and SGCs. With RAMP1 and CTR double immunohistochemistry, it was shown that co-expression exists in neurons (short arrows), SGCs (arrow heads), and fibers (long arrows), indicative of AMY_1_ receptor expression. RAMP3 is expressed in the nuclei, both neuronal and glia cell nuclei, and in addition in some SGCs. CLR is expressed in neurons, in the SGCs, and the thick fibers. Double immunohistochemistry shows co-expression in the SGCs (arrow heads), which suggests a presence of AM_2_ receptor

RAMP1 was expressed in neurons, in the SGCs and the Aδ fibers. CTR alone was expressed with varying intensity in most neurons and SGCs. RAMP1 and CTR double immunohistochemistry showed co-expression in some neurons, SGCs and fibers, which indicates AMY_1_ receptor expression.

RAMP3 and CTR double immunohistochemistry revealed a possible AMY_3_ receptor in the cytoplasm of the SGCs.

RAMP3 is expressed in the nuclei, both neuronal and SGCs nuclei. CLR is expressed in the cytoplasm of neurons, in the SGCs and the Aδ fibers. Double immunohistochemistry showed co-expression in the SGCs, which suggests a presence of AM_2_ receptor.

Double immunohistochemistry using combinations between CLR, RAMP2, and CTR antibodies could not be performed since all three antibodies were made in rabbit.

### Ligand/Receptor Expression (Fig. 6)

The ligands are expressed in different manners: CGRP and CT are expressed in neurons and in pearl-like fibers. In addition, SGCs are CT immunoreactive. AMY was exclusively expressed in the neurons. AM was expressed in the glial cells.

**Fig. 6:**
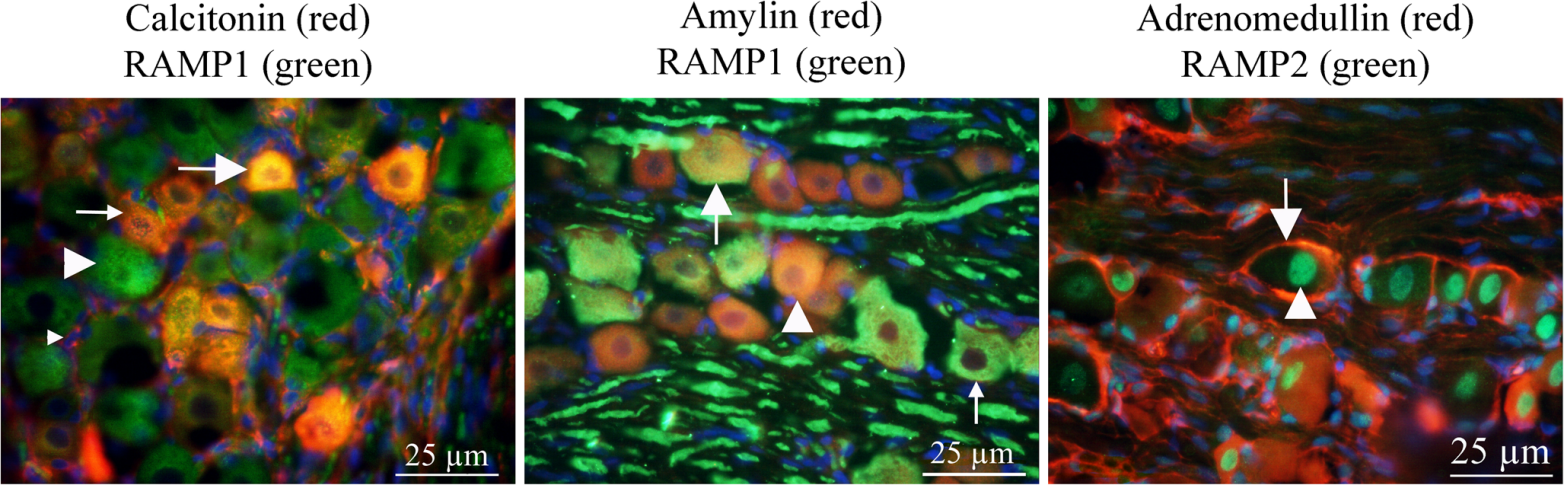
Ligand/receptor immunohistochemistry CT and RAMP1 double staining revealed co-localization in some neurons (long arrow). However, some neurons were only CT immunoreactive (thin arrow) and some only RAMP1 immunoreactive (arrow head). The same pattern of immunoreactivity was found using AMY and RAMP1 antibodies; some neurons showed co-localization (arrow), some AMY (arrow head), and some RAMP1 immunoreactivity (thin arrow). AM is expressed in the glial cells (arrow) and RAMP2 in the nuclei (arrow head); consequently, no co-localization was found using double immunohistochemistry

The receptor components show the same diversity in their pattern: CLR and RAMP1 immunoreactivity in the neurons, in the SGCs, and in the thick fibers; RAMP2/3 in the neuron; and SGC nuclei and CTR in most neurons and SGCs.

We have previously shown that no co-localization exists between CGRP and CLR/RAMP1 (Eftekhari et al. 2010). We confirm the results in the present paper (data not shown).

Double CT and CTR immunohistochemistry showed that all CT immunoreactive cells also were CTR positive (data not shown). No co-localization was found in the fibers (CT positive) or in the glial cell nuclei (CTR positive).

CT and RAMP1 double staining revealed co-localization in some neurons. However, some neurons were only CT immunoreactive and some only RAMP1 immunoreactive. The same pattern of immunoreactivity was found using AMY and RAMP1 antibodies; some neurons showed co-localization, some AMY, and some RAMP1 immunoreactivity.

AM is expressed in the glial cells and RAMP2 in the nuclei; consequently, no co-localization was found using double immunohistochemistry.

### Distribution of the CGRP Family of Peptides and their Receptors (Fig. 7)

The figure describes, in a schematic drawing, the simplified distribution of the CGRP family of peptides and their receptors. The CGRP receptor (CLR/RAMP1) is mainly expressed in the large neurons and SGCs, the adrenomedullin_2_ receptor (CLR/RAMP3) in some SGCs, the amylin_1_ receptor (CTR/RAMP1) in large neurons and SGCs, and the amylin_3_ receptor (CTR/RAMP3) in some SGCs.

**Fig. 7:**
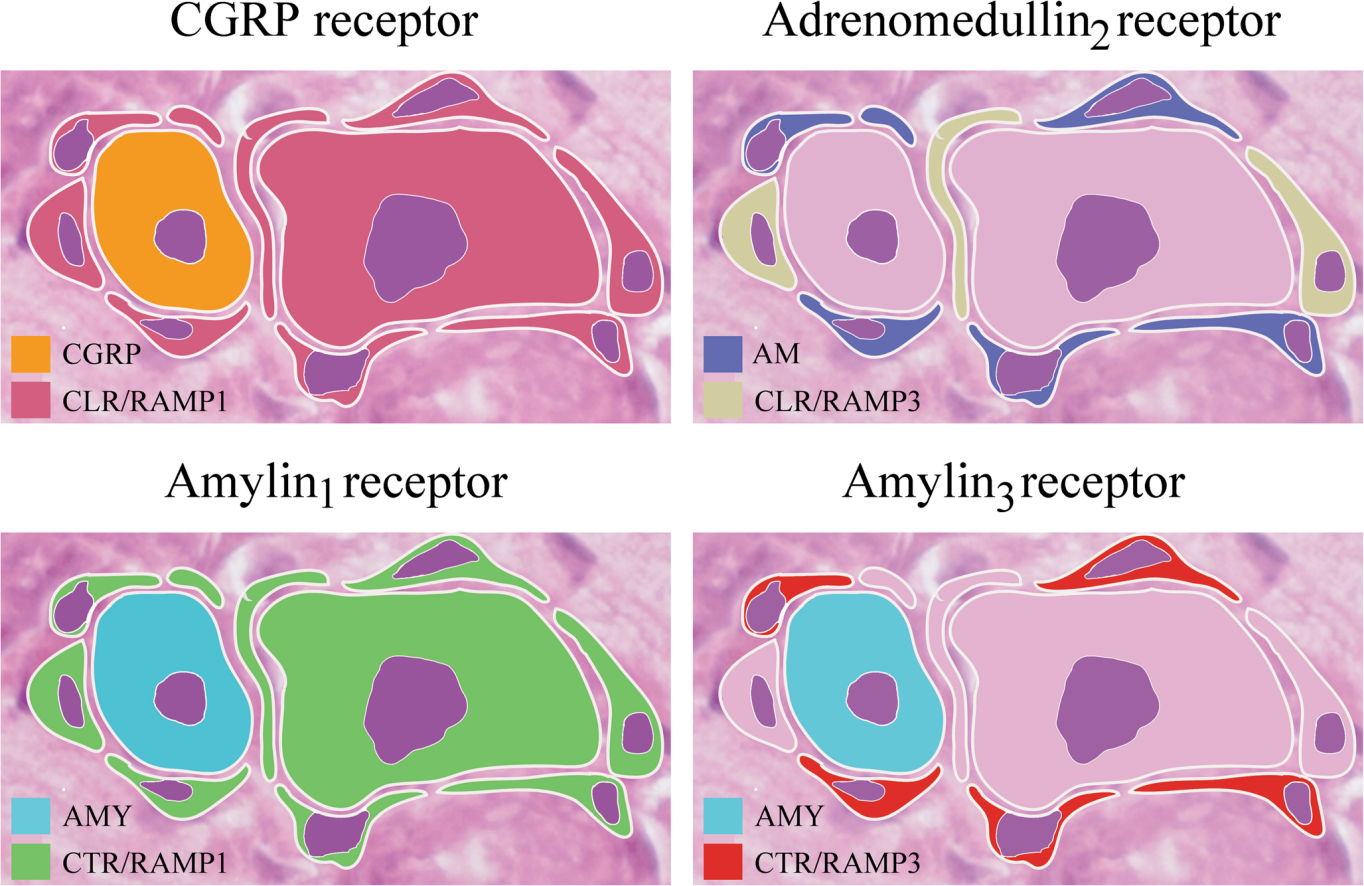
Schematic drawing of receptors. The CGRP receptor (CLR/RAMP1) is mainly expressed in the large neurons and SGCs, the Adrenomedullin_2_ receptor (CLR/RAMP3) in some SGCs, the Amylin_1_ receptor (CTR/RAMP1) in large neurons and SGCs, and the Amylin_3_ receptor (CTR/RAMP3) in some SGCs

### RAMP1 Immunohistochemistry of TG Neuron and Cerebellar Purkinje Cell (Fig. 8)

To give an example on how the cellular distribution of the different receptor components may vary, we compared the expression of RAMP1 in a TG neuron with that of a cerebellar Purkinje cell. The TG neuron expresses RAMP1 in the cytoplasm and the Purkinje cell on the cell surface (results previously published by (Edvinsson et al. 2011; Eftekhari et al. 2010)).

**Fig. 8:**
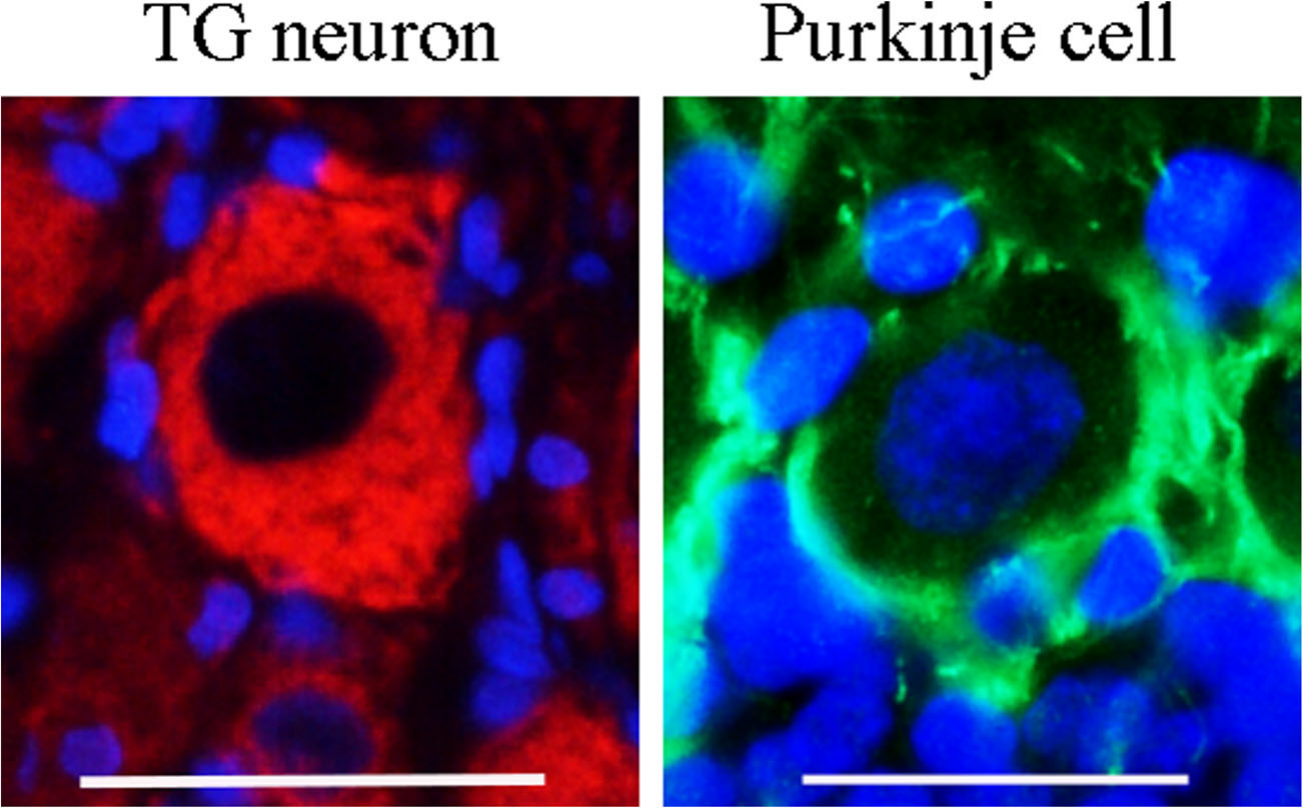
RAMP1 immunohistochemistry of TG neuron and cerebellar Purkinje cell. The left image shows a large neuron of rat TG and the right image a rat cerebellar Purkinje cell, using RAMP1 immunohistochemistry. The TG neuron displays RAMP1 staining in the cytoplasm and the Purkinje cell the cell surface. Scale bars 25 μm

### Quantitative PCR (Fig. 9)

Levels of mRNA expression for adrenomedullin, amylin, CLR, RAMP1, RAMP2, RAMP3, and CTR were measured using qPCR. The experiments confirmed the expression of all the genes except CTR (Ct values > 35, see dotted line) indicating little or no gene expression of CTR in TG.

**Fig. 9:**
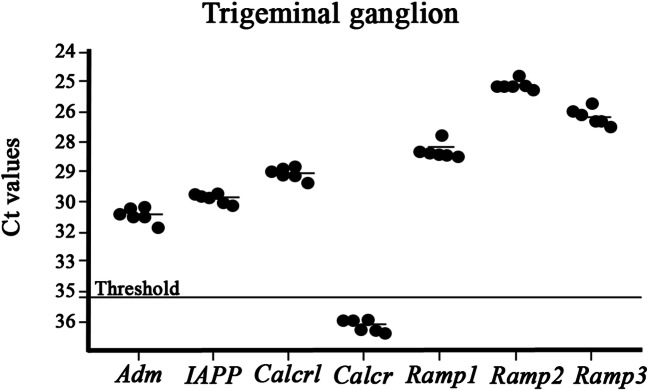
qPCR experiments confirmed the expression of mRNA for adrenomedullin (*Adm*), amylin (*IAPP*), CLR (*Calcrl*), RAMP1 (*Ramp1*), RAMP2 (*Ramp2*), and RAMP3 (*Ramp3*) in rat TG. The experiment confirmed the expression of all the genes except CTR due to the expression above the limit of detection (Ct values > 35, see dotted line). Values are expressed as mean ± SEM and *n* = 6

## Discussion

This is the first systematic and in detail comparison of the localization and expression of the CGRP family of peptides and their receptors in the trigeminal system. Each of the peptides of the CGRP family exhibits a distinct selection of biological actions (Poyner et al. 2002). CGRP and AMY are the most closely related peptides in terms of amino acid sequence which may cause an overlap in the ability to activate each receptor. Both CGRP and AMY are reported to have effects relating to pain, although, there is still limited data, and it is unclear how much overlap there is, because the peptides are not usually tested in the same study (Iyengar et al. 2017). The receptors themselves are also related and share components (Table 1). The relative potency of CGRP, CT, AMY, or AM to the different receptor complexes is a complicated topic, for example CGRP > AM to RAMP1/CLR, CT > AMY to CTR. Their functional cross-reactivity makes the work challenging (Hay et al. 2018; Hendrikse et al. 2018).

Within the CGRP family of peptides, the N-terminus and C-terminus are the most highly conserved regions with more divergence in the mid-portion of each peptide suggesting an importance in retention of the N-terminal and C-terminal for biological activity (Bower and Hay 2016). The receptor component CTR was cloned in 1991 (Lin et al. 1991), CLR in 1993 (Njuki et al. 1993), and RAMP1 was discovered in 1998 (McLatchie et al. 1998). A sequence database search for expressed sequences similar to RAMP1 identified RAMP2 and RAMP3 (Naot and Cornish 2008).

It is clear that there is a complex interplay among the ligand and receptors, which acquire an experimental system to allow the results to be further validated. However, the examinations in the present study describing the distribution and mRNA levels of the markers might define potential targets of different pathological conditions. So far, immunohistochemical examination of the CGRP family of peptides and their receptors in TG has been performed with CGRP and its receptor components CLR and RAMP1 (Eftekhari et al. 2010). In addition, AMY has been demonstrated in rat TG (Walker et al. 2015). Moreover, Tajti and colleagues found that the SGCs in TG contained Pro-CT which is the pro-peptide form of calcitonin prior splicing to CGRP in neuronal tissue (Tajti et al. 2011). We confirm these results in the present study (Fig. 2b). To our knowledge, immunohistochemistry using RAMP2 and RAMP3 antibodies has hitherto not been shown in the TG or the CNS, except for the rat retina (Warfvinge and Edvinsson 2019b). It is important to compare the peptides side-by-side to help to address questions of which receptor(s) mediate the effects and which are actions as a result to cross-reactivity between receptors. The analysis of the expression of the peptides and the receptors with qPCR revealed their quantitative expression of respective mRNA (Fig. 9). These results support the immunohistochemistry results which provide cellular localization.

The CGRP family of peptides are ligands for closely related family B of G protein-coupled receptors (GPCRs) and share structural homology (Hay et al. 2014). Class B GPCRs are involved in major biological and pathophysiological functions (Hoare 2005). It is now clear that RAMPs can interact with a wider range of GPCRs, e.g., all three RAMPs can interact with the VPAC1 (vasoactive intestinal polypeptide/pituitary adenylate cyclase-activating peptide) receptor. In addition, RAMPs can produce a number of different effects on ligand binding, signal transduction, and receptor trafficking (Hay and Pioszak 2016). Furthermore, Walker and colleagues stated that the results obtained by a GPCR array identified many additional GPCRs within the TG as potential pharmacological targets for migraine which have been previously appreciated (Walker et al. 2015).

### Calcitonin Gene-Related Peptide

Antibody treatments towards CGRP itself and towards the CGRP receptor have been approved by regulatory bodies (FDA and EMA) and have reached the clinic as remedies against frequent episodic migraine and chronic migraine (Diener 2019). There are three monoclonal antibodies towards the ligand CGRP and one directed towards the C- and N-terminals of the CGRP receptor. The clinical results have so far been excellent with good efficacy and minor side effects (Edvinsson et al. 2018). Overall, 20% of the patients are super responders (end of headache) while approximately 50% show considerable reductions in migraine headache. The remainders do not respond, and here, we might hypothesize that other molecules or receptors are involved such as amylin.

Since the antibodies do not pass the blood-brain barrier, the efficacy is thought to occur at some parts of the trigeminal system (Lundblad et al. 2015). Importantly, about half of the neurons in the TG store CGRP, and the remainder store CLR/RAMP1, suggesting that CGRP is the key player. CGRP is expressed in many neurons, mainly in small to medium-sized neurons (Figs. 2 and 4). The cellular CGRP is often expressed in or close to the membranes of the Golgi apparatus (Warfvinge, unpublished). In addition, pearl-like CGRP immunoreactivity was detected in fibers that are of the C-type of sensory unmyelinated nerves. The myelinated fibers do not contain CGRP. The receptor components CLR and RAMP1 are expressed in neurons (mainly the larger ones), the SGCs and the thick fibers (typical in Aδ-fibers) (Figs. 3, 5, 6, and 7). CGRP, CLR, and RAMP1 distribution in rat TG have earlier been examined in details (Eftekhari et al. 2010; Lennerz et al. 2008; Miller et al. 2016). The present paper has confirmed these results and extends details of the expression.

Walker and colleagues demonstrated that CTR is expressed in small- to medium-sized neurons and some larger neurons in TG using immunohistochemistry (Walker et al. 2015). In addition, the authors showed that CTR and RAMP1 expressing neurons co-localized. This is in accordance with our results in the present paper using immunohistochemistry. However, by using qPCR, the mRNA expression of the CTR gene was above the limit of detection. The inability to detect the CTR gene by qPCR could be explained by a problem with the reliability of the antibody used. Still, it has been demonstrated that existing incongruent expression between mRNAs and protein can only be unveiled through integrated analyses of both proteins and mRNAs, i.e., to examine more than one aspect of a biological system (Tian et al. 2004).

AM has affinity for CGRP receptors, and CGRP to AMY_1_ receptors (Hendrikse et al. 2018) (Table 1). AMY and CGRP are the most closely related peptides in terms of amino acid sequence which may cause an overlap in the ability to activate each receptor. Also, the receptors themselves are related and share components: CLR/RAMP1 (ligand CGRP) and CTR/RAMP1 (ligand AMY). Given the close relationship between AMY and CGRP, since they share the AMY_1_ receptor (CGRP = AMY, Table 1), it is worthwhile considering whether amylin is able to trigger migraine. However, the CGRP receptor antibody Erenumab is made to target the N-terminals of CLR and RAMP1, which makes it very specific for the CGRP receptor. The CGRP ligand antibodies do not have such specificity; in fact, they target both αCGRP and βCGRP. Despite this, the antibodies are equally effective.

### Adrenomedullin

AM is chiefly found in endothelial cells and was first isolated in 1993 (Kitamura et al. 1993). AM has been found to be generally expressed and to participate in a variety of physiological functions including vasodilation, bronchodilation, growth, and hormone regulation (Ferrero et al. 2018b). Furthermore, AM is involved in several pathophysiological processes such as hypertension, retinopathy, and tumor genesis (Ferrero et al. 2018a).

In 1994, active production of AM in cultured endothelial cells was demonstrated (Sugo et al. 1994). In mammals, endothelial AM immunoreactivity has been inconsistently reported. The reason for this might be that AM in the vascular endothelium is present in low concentrations (Satoh et al. 1996). AM has also been localized in neurons and glial cells (Serrano et al. 2000). In the present study on rat TG, AM was expressed in the glial cells, both the SGCs and cells enveloping the neuronal processes, probably myelinating Schwann cells (Figs. 2 and 6). In addition, immunoreactivity was found in blood vessel walls indicating vascular endothelial staining. Interestingly, immunohistochemistry on rat retina using the same antibodies as reported here, only the vasculature in the inner part of the retina displayed AM immunoreactivity (Warfvinge and Edvinsson 2019b).

We performed double immunohistochemistry with ligand to ligand and ligand to receptor. No co-localization was found between AM and the other members of the CGRP family, even though overlap is known to occur because the peptides share features. The AM receptors consist of CLR and RAMP2 (AM_1_ receptor) and CLR and RAMP3 (AM_2_ receptor) (Table 1). Double immunohistochemistry with AM and RAMP2 (AM receptor component) antibodies showed no co-localization; AM is mainly expressed in the glial cells and RAMP2 in the nuclei; consequently, no co-localization was observed. The gene expression of CLR, RAMP2, and RAMP3 are all present in the TG. Receptor/receptor immunoreactivity with RAMP3 and CLR showed weak double staining in the glial cells, mainly SGCs (Fig. 5). However, relatively low expression does not automatically translate into little function.

In a study by De Martin and colleagues, the expression of AM and its receptor components in human thymic tissue from newborns were investigated. They demonstrated that AM and RAMP2, but not RAMP3, were largely distributed within the human thymus. RAMP2 was expressed both in the cytoplasm and in the nucleus. They concluded that the AM_1_ receptor has an intracellular localization and suggested that AM exerts a control of its functions through the interaction with the receptor component localized in the nucleus (De Martin et al. 2014). We confirm the presence of RAMP2 and RAMP3 in the nuclei of rat TG cells. AM might exert control of its function also in rat TG through interaction with RAMP2 and RAMP3 in the nucleus.

To summarize, we suggest that AM is present in the glial cells and in the vasculature of rat TG. Furthermore, we suggest that an AM functional receptor (AM_2_) exists in mainly the SGCs of rat TG. Interestingly, AM did not show any migraine-like effect upon systemic administration in man (Ashina et al. 2017).

### Calcitonin

CT was discovered more than 40 years ago (Copp and Cheney 1962) and is a hormone produced by C cells of the thyroid, whose role is to reduce plasma calcium levels and to promote bone formation (Findlay and Sexton 2004). CT is used clinically in the treatment of bone disorders characterized by increased bone resorption, osteoporosis, and hypercalcemia due to malignancy (Findlay and Sexton 2004). However, there is still yet much to learn about the actions and role of CT, in particular to migraine-related structures.

The presence of CT and its receptor in a large number of cell types and tissues suggests multiple physiological roles (Findlay and Sexton 2004). The mediated actions of CT correlate well with the location of CT binding sites. Binding of CT to a specific receptor induces morphological changes in osteoclasts, which results in bone resorption (Chambers and Moore 1983). CT has not been shown to be expressed in the nervous system; however, binding sites for CT are found in many brain structures (Hendrikse et al. 2018). The present study shows CT immunoreactivity in a similar pattern as for CGRP; CT expression in vesicles and pearl-like CT immunoreactivity in C-type of sensory fibers. Hence, we show for the first time that CT is expressed in the nervous system.

The CT receptor CTR acts by itself as a specific CT receptor by reaching the cell surface (Hay et al. 2018). In the present study, it was shown that CTR is expressed in most neurons, SGC, and glial cell nuclei with varying intensity. Although, there was an extremely little or no gene expression of CTR in the TG as well as there was no indication of CTR being present on the cell surface and thereby no indication of activated CTR. However, the expression of CTR resembled that of CLR/RAMP1, the well-documented and recognized CGRP receptor (Hay et al. 2018), and both CLR and RAMP1 are expressed at mRNA levels in TG. To our knowledge, CLR/RAMP1 immunoreactivity has not been shown at cell surfaces of TG. However, we have previously described the distribution of CGRP and its receptor components in rat cerebellum (Edvinsson et al. 2011), where we found the CGRP receptor on the surface of the Purkinje cells (Fig. 8). If this is an effect of functionality and that intracellular CLR/RAMP1 distribution in rat TG is a consequence of no function is not known. However, the literature shows that the CLR/RAMP1 receptor in the TG is a functional receptor even though the distribution of this receptor is not expressed on the surface but intracellularly (Eftekhari et al. 2010). We suggest that the intracellular expression of CTR, as CLR/RAMP1, in rat TG denotes a functional CT receptor, and that CT exerts control over its function through interaction with CTR.

mRNA profiles are often used as surrogates for protein expression. However, mRNA expression can be used to explain at most 40% of the protein expression which is likely a reflection of the underlying biological mechanisms (Tian et al. 2004). We found that the expression of CTR mRNA was above the detection limit (Ct > 35), which could indicate little or no gene expression of CTR in TG. The dissimilar expression between CTR protein and CTR mRNA may be a result of biology of gene expression rather than measurement errors.

### Amylin

Human AMY was probably first observed in 1901, and described as hyaline deposits in pancreas of patients with type 2 diabetes (Opie 1901). AMY was isolated in 1987 (Cooper et al. 1987), and it is an endocrine hormone that signals to the brain and acts as a satiety factor. Possibly, AMY may also have other roles (Hay 2017). Research on AMY neuronal deposition has mainly focused on its role in Alzheimer’s disease (Mietlicki-Baase et al. 2017). Recently, it was shown that AMY alters human brain pericyte viability (Schultz et al. 2017). In addition, AMY immunoreactivity has been described in several places along the gut and in some neurons (Hay 2017; Young 2005). We have reported AMY in the TG of cat and that AMY can dilate in vitro as well as in vivo cerebral vessels (Edvinsson et al. 2001). The gene is also expressed in TG.

Walker and colleagues showed the presence of CTR, and furthermore, they suggested that the AMY_1_ receptor (CTR/RAMP1) could be a relevant target for CGRP and perhaps a novel way in migraine therapy (Walker et al. 2015). We support the relevance of AMY_1_ being a migraine therapy target. In the present study, we show that RAMP1 is expressed in neurons, in the SGCs and the thick fibers (Fig. 5). Furthermore, CTR is expressed with varying intensity in most neurons and SGCs. RAMP1 and CTR double immunohistochemistry showed co-expression in some neurons, SGCs, and fibers, indicating an AMY_1_ receptor expression. RAMP3 and CTR double immunohistochemistry revealed a possible AMY_3_ receptor in the SGCs.

Findings on thymic epithelial cells using both immunofluorescence and immunogold stainings demonstrated RAMP2 localization in the nucleus, CLR both intracellularly and in the plasma membrane and AM in the cytoplasm. These results agree with our results. The authors suggest that AM activates its receptor in the nucleus to modulate transcription (Castellani et al. 2016). Furthermore, recent finding by Castellani on AM and its receptor distribution strengthens our results and indicates that absence of co-localization does not necessarily mean absence of functional receptors. Moreover, it is possible that the expressional profile might differ in disease (Saeed et al. 2009). Future studies in animal models might unravel unexpected expressional profiles and indicate ways for therapy.

## Conclusion

It is important to compare peptides and receptors side-by-side to help address questions of actions resulting from cross-reactivity between receptors. We demonstrate in the present study using qualitative and quantitative methods that calcitonin is expressed in the rat nervous system, i.e., the TG and that possible AM_2_, AMY_1_, and AMY_3_ receptors occur.

Several of the diverse biological actions of the CGRP family of peptides are clinically relevant. Our findings demonstrate the specific ligand and receptor sites in the rat trigeminal ganglion, highlighting recognition mechanisms to facilitate drug development.
